# The Rise of Tech Ethics: Approaches, Critique, and Future Pathways

**DOI:** 10.1007/s11948-024-00510-3

**Published:** 2024-10-09

**Authors:** Nina Frahm, Kasper Schiølin

**Affiliations:** School of Communication and Culture, Department of Digital Design and Information Studies, Aarhus University, Aarhus, Denmark

**Keywords:** Ethics, Technology, Controversy, Imaginaries, Governance, Critique

## Abstract

In this editorial to the Topical Collection “Innovation under Fire: The Rise of Ethics in Tech”, we provide an overview of the papers gathered in the collection, reflect on similarities and differences in their analytical angles and methodological approaches, and carve out some of the cross-cutting themes that emerge from research on the production of ‘Tech Ethics’. We identify two recurring ways through which ‘Tech Ethics’ are studied and forms of critique towards them developed, which we argue diverge primarily in their a priori commitments towards what ethical tech is and how it should best be pursued. Beyond these differences, we observe how current research on ‘Tech Ethics’ evidences a close relationship between public controversies about technological innovation and the rise of ethics discourses and instruments for their settlement, producing legitimacy crises for ‘Tech Ethics’ in and of itself. ‘Tech Ethics’ is not only instrumental for governing technoscientific projects in the present but is equally instrumental for the construction of socio-technical imaginaries and the essentialization of technological futures. We suggest that efforts to reach beyond single case-studies are needed and call for collective reflection on joint issues and challenges to advance the critical project of ‘Tech Ethics’.

## Introduction

As technological innovations are becoming ever more central to the ways societies imagine and pursue desirable futures, their potentially undesirable effects are increasingly moving to the center of public debates, governance considerations, and corporate strategies. The recent surge of AI technologies such as ChatGPT and heated controversies around their social implications is but the latest example in the turn to ‘Tech Ethics’ taking place across private and public settings, research and development, and various technoscientific domains since the beginning of the 21st century. While attention to ethical questions in emerging science and technology in the 20th century was largely concerned with biomedical research and experimentation, giving rise to ‘Bioethics’ as a powerful regime of governance, today’s rise of ‘Tech Ethics’ represents a new modality through which societies cope with the uncertainties of socio-technical change and the digital transformation in particular. Expanding well beyond tried and tested means of ethical deliberation such as ethics councils and institutional review boards, ‘Tech Ethics’ today reflects a heterogeneous landscape of actors, institutions, discourses, and tools involved in setting R&D and policy agendas, the development of frameworks and instruments for governance, and the transformation of scientific and engineering practice. From high-level commissions and forums launched by governments and international organizations, the hiring of tech ethicists and advisory boards in companies and labs, to the surge of civil society with a dedicated focus on tech or the creation of new academic institutes, coming to terms with ‘Tech Ethics’ constitutes a key ingredient of contemporary innovation politics.

This Topical Collection maps current approaches to ‘Tech Ethics’, identifies some of their deficits, and suggests a number of ways on how to better address the ethics of tech in the future. As detailed in the call for papers for the collection, contributions have been driven by questions such as, but not limited to: how do we make sense of the evocation of ‘Tech Ethics’ by different actors in and around innovation? Which new forms of ethical and social deliberation are being institutionalized in public and private, local and global governance settings? Which problems and deficits legitimize such initiatives to emerge? And to what, and to whom, are these new ethical aspirations, discourses, and practices responding? With backgrounds in science, technology, and society studies (STS), philosophy, history, law, and anthropology, contributions to the collection showcase how the rise of ‘Tech Ethics’ is observed, assessed, and scrutinized from various disciplinary perspectives. In this editorial, we provide brief summaries of the collection’s six papers and carve out some of their commonalities and differences, particularly with regard to their analytical approaches and the forms of critique of ‘Tech Ethics’ that can be derived from them. We conclude with reflections on how this collection might contribute to the evolving research agenda on ‘Tech Ethics’.

## Papers in this Collection

Grounded in Goffmann’s theory of metaphors and its uptake in STS, Huang and Krafft’s contribution to the collection, “Performing Platform Governance”, explores how Facebook (Meta) has responded to the increasing public demand for transparency in platform governance. They argue that while providing their users with more opportunities for authentication and privacy control at the ‘front-stage’, the recommendation and advertisement happening at the ‘back-stage’ remained hidden, yet still determines the exposed content and user experience. Understanding this strategic boundary drawing between ‘front’ and ‘back stage’, between what is rendered visible and invisible for the user, the paper suggests, is key for critique of the governance and ethical implications of social media platforms.

In “How Neurotech Start-Ups Envision Ethical Futures”, Knopf, Frahm and Pfotenhauer also identify the drawing of boundaries – or, in the vernacular of STS, boundary work – as a strategy of constructing ‘ethical realities’ by corporate actors. Based on fieldwork in direct-to-consumer neurotechnology start-ups, the paper analyzes, for instance, how actors draw boundaries between their products’ actual, practical risks and their hypothetical future risks, hence deferring some ethical issues to a future in which neurotechnologies are already widely disseminated and used. Boundary work here helps to delineate the governance obligations of start-ups towards the ethics of their products, forming part of the nascent ‘knowledge-control regimes’ that underwrite ethics discourses and practices in the field.

Smallman’s paper “Multi Scale Ethics” argues that that principles of transparency, fairness, responsibility and privacy, which have been dominant in constructing ‘AI Ethics’, widely neglect the manifold implications of AI and its use in healthcare in particular. Analyzing current guidelines for AI, the paper identifies an overt emphasis on AI’s impact on the individual rather than on society as a whole. To account for these wider social impacts, Smallman suggests a multi-scale ethics framework that considers how AI also shapes worlds at global, national, systems and institutional levels, as well as groups and communities. Assessments of AI technologies through the framework of multi-scale ethics do not only enable a holistic mapping of issues and impacts, but a thorough consideration of the dynamic relationship between technological and social systems.

Grellette’s contribution to the collection, “Tech Ethics Through Auditing”, departs from the premise that the public’s trust in the tech industry is dramatically waning and that many technology companies, in response, have committed to ‘tech ethics’. Paradoxically, this has only furthered the public’s distrust, as the media has increasingly drawn attention to the mobilization of ethics as mere acts of lip service. This, in turn, has led to delegating ‘tech ethics’ to actors outside the tech industry, such as the EU or national governments. The paper suggests an alternative approach, in which technology companies’ commitment to, and practices of, ethics are subjected to public trust audits, i.e. to continuous, community-based evaluations of the ethical legitimacy of tech companies and their products.

Collaboration and participation in the making of ethically acceptable AI in healthcare is also the topic of Gundersen and Bærøe’s contribution, “The Future Ethics of Artificial Intelligence”. Analyzing four models for designing medical AI, they suggest that public participation is particularly important when the technologies are imagined to fundamentally transform the conditions for ethical decision-making. In such cases, where AI technologies have the potential to disrupt medical practices and decision-making, the participation of AI designers, bioethicists and medical experts should be expanded to the participation of the general public and policy-makers to spur broad public debate on the costs and risks of the transformative technologies in question.

Whitman’s paper “Modeling Ethics” is based on long-term ethnographic fieldwork among developers, administrators and users of an app developed and disseminated by a large American public university to predict students’ academic success. Though resembling cutting-edge commercial educational technologies, the app is the result of a research project at the university, and as such subject to the local institutional review board (IRB). The paper, however, shows that the IRB is not modeled to capture the various circumstances of the students’ consent, particularly the app’s reuse of data collected for other purposes. Whitman conceptualizes this repurposing of data that escapes the IRB’s jurisdiction as ‘data creep’ and concludes the paper with reflections on how big data ethics could be remodeled around refusal rather than consent in order to better mitigate the problems of data creep.

As the diverse contributions to this collection evidence, ‘Tech Ethics’ are not only situated in a multiplicity of settings, technoscientific domains, and actor constellations but the study of ‘Tech Ethics’ in the social sciences and humanities is heterogeneous in itself, rendering attempts at delineating its boundaries a challenging endeavor. Yet, in the remainder of this editorial, we attempt to draw some of the contours that emerge from analysis and critique of ‘Tech Ethics’ in the collection’s contributions: (1) two recurring yet different analytical angles through which the production and performance of ethics in tech is approached, (2) shared findings regarding the relationship between technological crises and the mobilization of ‘Tech Ethics’, and (3) the important role for ethics in legitimizing contemporary investments in innovation and the corollary making of technological futures.

## Two Approaches to ‘Tech Ethics’

The six contributions assembled in the collection can be grouped around two recurring ways of approaching ‘Tech Ethics’ in the social sciences and humanities today. On the one hand, we find those reading current discourses and practices of ethics in tech development and governance through attending to a set of normative commitments and principles in their analysis of and intervention in ‘Tech Ethics’ – for instance, to shared decision-making (Gundersen & Barøe, 2022), trust (Grellette, 2022) or a rejection of individualist ethical principles and forms of implementation (Smallman, 2022). On the other hand, we find explorations of ‘Tech Ethics’ as an empirical field and actor’s category to be critically examined by research, for example through asking who is involved or excluded in settling the ethics of tech, how ‘Tech Ethics’ is enacted and performed by organizations and individuals, and what kinds of frames are mobilized in the production and proclamation of ‘Tech Ethics’ (Knopf et al., 2023; Huang & Krafft, 2024; Whitman, 2021). While both depart from observing a novel role for anticipating, addressing and attending to questions concerning the moral and normative dimensions of emerging technoscience, differences in disciplinary and methodological approaches also imply the identification of different problems and articulations of critique towards the turn to ‘Tech Ethics’ in research.

A first group of studies in this collection identifies current ethics strategies, practices and policies as widely deficient in their attention to ‘wider sociological concerns’ regarding technological innovation (Smallman, 2022) or ‘ethical value judgements in both design and application’ (Gundersen & Barøe, 2022). Critique derived from these analyses revolves around institutionalized ethics discourses and rationales that favor narrow framings of the effects of technologies on the individual, a division of labor among tech developers, experts and users, or the mere ‘ethics-washing’ of organizational practices and products for the sake of ever greater profit accumulation. These forms of critique are, at the same time, productive of new suggestions for how to overcome current deficits in ‘Tech Ethics’ and, indeed, for innovating the ways the ethics of innovation are pursued today.

As various contributions to the collection illustrate, such critical engagement with ‘Tech Ethics’ can serve as much as a tool to identify current problems and challenges in the pursuit of ethical innovation and as a rich source for new forms of conceiving what the ethics of innovation are and how they ought to be put into practice by actors in an innovation system. Grellette, for example, proposes a new form of trust auditing for companies through “community-based evaluations of technology developers’ ethical trustworthiness” (2022). Gundersen and Barøe suggest that principles of shared decision making in tech development and application are best pursued through collaborative and participatory models that connect different actors and their competencies (2022). And Smallman’s multi-scale ethics framework enables assessment of the impact of a technology at different scales, from the individual to the global and across time, “to account for the possibility that the effects of technologies can look very different from different standpoints; that the risks and benefits of technologies are often uncertain, tend to pattern; and that the ethical impacts include profound effects on social, institutional and democratic arrangements” (2022).

The other group of approaches follows the actors, practices and discourses involved in the current turn to ‘Tech Ethics’ and considers them as empirical and performative sites to be deconstructed by the analyst without explicit a priori normative commitment to a set of ethical norms and principles. Through ethnographic observations and interviews with developers and users of new technologies, these analyses carve out, for instance, how ethics are modeled through the enactment of institutionalized scripts as well as through practices conceived as ‘doing good’ in particular contexts and situations (Whitman, 2021), how the pursuit of ethics is envisioned to inform and engender desirable technological futures (Knopf et al., 2023), or how the ethics of technology are relegated to specific technological features and functions at the expense of others (Huang & Krafft, 2024). Rather than the identification of deficits in ethical frames and practices through the analyst, they foreground the ways actors in diverse domains of technological application and development draw boundaries between ethical/unethical innovation, construct and delegate authority over settling and pursuing ethics, or frame technological vanguard projects and responsibility for their ethics.

Yet contributions within this second group are not limited to merely describing different forms of ethics construction and performance across various technological domains. By generating empirically informed and ethnographically thick accounts of ‘Tech Ethics’, they allow better understanding – and critique – of the role of ethical discourse and practice in the creation of legitimacy, credibility, and authority for certain technological pathways and with them, particular forms of social order. Knopf and colleagues, for example, identify “two forms of displacement—the ‘deferral’ of ethical challenges and benefits to the future, and the ‘delegation’ of ethical reasoning to established knowledge regimes of ethical oversight” in the present, allowing neurotech start-ups “to construct plausible and desirable technology trajectories” by shaping a specific understanding of ethics and responsibility in technological development and deployment (2023). Huang and Krafft, in turn, reveal the front- and backstage data relations constructed by Facebook that enable modifications of data use “while obscuring the information flows central to the economic value of the platform” (2024). And in Whitman’s study of data creep in higher education, models of ethics “leave the constitution of ethics up to institutions and individuals that are not set up to confront data ethics issues as larger systemic asymmetries in power, either because, respectively, they maintain it or cannot maneuver through structural barriers and constraints”, precluding “possibilities for asking bigger questions about what ethics are and could be” (2021). Critique derived from analyses such as these then informs proposals for re-configuring current power relationships that shape the making of ‘Tech Ethics’, for a collective re-modeling of ethics production, and for greater reflexivity towards frames and rationales informing the development of ethics *and* technology.

## Tech (Ethics) under Fire

Beyond these broader differences, contributions to this collection evidence how the turn to ‘Tech Ethics’ is closely related to contemporary public controversies and debates regarding the social implications of technoscientific innovation, particularly in the domain of digital technologies and AI. Not only have words such as ‘Techlash’ increasingly shaped the vocabulary through which technological controversies have been taken up across countries, arguably representing a “growing public animosity” towards Big Tech and its products (Foroohar, 2018). Various scandals around the ways Tech Ethics has been institutionalized (Metcalf et al., 2019), transformed (Phan et al., 2021) and ‘invented’ (Ochigame, 2019) by the Tech industry in response to such backlash point to a crisis of ‘Tech Ethics’ in itself, which today faces heightened public attention and scrutiny as yet another strategy of corporate capture and power. Recently, the credibility and legitimacy of mobilizations of ‘Tech Ethics’ in public sector innovation policies and regulatory initiatives has similarly come under fire, particularly with regard to the participation of corporate lobbies in expert groups and commissions tasked with writing ethics guidelines for AI (e.g. Corporate Observatory Europe, 2023) or regarding the “ethification” of public instruments for regulating digital technologies at the expense of stricter legal accountability regulation (e.g. van Dijk et al., 2021).

These interrelated crises – of technological innovation and its pursuit, and of the ethics of ‘Tech Ethics’ (Hagendorff, 2020) – play out in various ways across the studies gathered in this collection. A common finding of contributing authors is that revelations such as Cambridge Analytica (Huang & Krafft, 2024) or controversial attempts at scaling up emerging technologies such as AI in public services (Smallman, 2022) fuel public protests and forms of resistance which are often countervailed through the production and performance of ‘Tech Ethics’, including through emphasis on the need for ‘trust’ among policy-makers, providers and users of technologies (Grellette, 2022), for collaboration and participation in tech development and governance (Gundersen & Barøe, 2022), or for adherence to existing ethical codes and scripts (Whitman, 2021).

‘Tech Ethics’ thus pertains to a wider shift towards what has been described as the construction of “social fix” logics in contemporary discourses around technoscience – i.e., the increasing importance of social inclusion and normative deliberation in the legitimization of innovation imperatives and for saving technological projects and agendas in crisis (Frahm & Doezema, 2021, Doezema & Frahm 2023a). As various contributions illustrate, such role is demonstrated by different processes set in motion through the production of ‘Tech Ethics’, such as closing down public debates, normalizing technologies and governance regimes, maintaining existing power relations or delegating responsibility and accountability. In all of these cases, ‘Tech Ethics’ figures as an instrumental response to the problems generated by technoscientific knowledge, products, and modalities for their governance, yet one which may quickly generate critique and gain legitimation issues itself. In this sense, ‘Tech Ethics’ is contingent, representing both a solution and a problem for the politics of innovation in the 21st century.

## Tech Ethics’ Future Essentialism

While discourses and practices of ‘Tech Ethics’ play a key role for technoscientific development and governance in the present, they also have an important function for anticipating and addressing potential social issues and public backlash in the future, and hence for producing the visions through which certain technologies come to be imagined as worthwhile of pursuit. Uncertainty underwriting innovation pathways demands from actors involved in research, development and policy to engage significantly in the construction of visions, expectations and imaginaries that re-assure citizens, users and consumers of technological products of the desirability of technological experiments and investments (Doezema, 2023). As shown by several contributions to this collection, it is through the invocation of ‘Tech Ethics’ and corollary commitments to good governance that technological futures are rendered safe for society and, indeed, are framed as ethical imperatives for societies to pursue.

This future essentialism at play in the current turn to ‘Tech Ethics’, in which “a fixed future […] serve[s] as a disciplining moral guideline in an otherwise messy and uncertain world” (Schiølin, 2019), is particularly visible in nascent technoscientific fields that have not yet scaled across society but is also an important feature for maintaining legitimacy of already widely disseminated technological products. In start-ups driving emerging consumer neurotechnology, for instance, “ethics figures as an element that contributes to mediating […] visions of successful technologies and desirable futures – and make them acceptable, plausible and reasonable” (Knopf et al. 2023). In Big Tech companies such as Facebook, frameworks such as ‘human centered design’ allow to “foreclose governance of the political economy of platforms” and its information asymmetries (Huang & Krafft, 2024). And in specific domains such as healthcare, AI ethics guidelines issued by expert committees and policy makers are “underpinned by an assumption that ethically-sound AI in healthcare is possible” (Smallman, 2022).

An equally important ingredient of the dialectic of future essentialism is the production of scenarios of runaway technology and unchecked technological development through'Tech Ethics' (Doezema & Frahm, 2023b). As Knopf et al. argue (2023), mobilizing ethics to counter such futures can serve to draw authoritative boundaries between good and bad, desirable and undesirable technologies and to shift certain ethical responsibilities and obligations to the hypothetical future of technological innovation. Once positioned on the ethically benevolent side of this dialectic, technological futures become framed as inevitably beneficial and hence desirable for society. As such, ‘Tech Ethics’ forms a crucial ingredient of the politics of expectations (Borup et al., 2006) and the consolidation of socio-technical imaginaries (Jasanoff & Kim, 2009) but can also work as a powerful tool for their contestation and transformation.

## Future Pathways for the Study of ‘Tech Ethics’

With the steady increase of public controversy and growing demand for greater social control and regulation of emerging technologies, ‘Tech Ethics’ forms an important site for both intervening in and criticizing the pursuit of science and technology in the name of social progress and wellbeing. As this collection illustrates, scholarship in the social science and humanities concerned with ‘Tech Ethics’ provides a number of different approaches and analytical angles for research, and has not run short in identifying persisting challenges in the ways ‘Tech Ethics’ are mobilized. While such heterogeneity reflects the diversity of situated ways through which ‘Tech Ethics’ are made and their variegated effects on technoscientific pathways and governance trajectories, cross-cutting issues and themes are rarely identified (Green, 2021).

This lack of synthesis among research calls for greater exchange among scholars with shared interests and motivations, as well as wider conceptualizations and theorizing on the role, function, and wider societal impact of ‘Tech Ethics’ in contemporary innovation politics. Reaching beyond single case studies and settings in which ‘Tech Ethics’ play out is key for a more systematic critique of the construction and impact of ethical norms, principles and practices in technoscientific world-making. This includes accounting for recent transformations in imperatives “from more innovation to better innovation” (Pfotenhauer, 2023) that inform the “new spirit of technoscience” (Doezema & Frahm, 2023a) and in which the turn to ‘Tech Ethics’ forms a crucial instrument and site to be studied.

Several themes emerging from the contributions to this collection could yield generative, cross-disciplinary discussions among scholars in the future. Amongst others, they include (1) the framing of the relationship between ethics and technology in actor’s discourses and through the scripts guiding institutional and organizational practice; (2) the boundaries drawn between socially desirable/undesirable technology; (3) the visions and imaginaries guiding the production of and reasoning on ethics and their essentializing effect; and (4) the construction of ethical deficits and solutions and their relationship to wider crises of innovation imperatives. Collective reflection on these themes and beyond can not only advance the critical project of ‘Tech Ethics’ but pave the way for a more sound and nuanced ethics of technology.
